# Identification of a 4-microRNA Signature for Clear Cell Renal Cell Carcinoma Metastasis and Prognosis

**DOI:** 10.1371/journal.pone.0035661

**Published:** 2012-05-18

**Authors:** Xiwei Wu, Lihong Weng, Xuejun Li, Chao Guo, Sumanta K. Pal, Jennifer M. Jin, Yuping Li, Rebecca A. Nelson, Bing Mu, Susan H. Onami, Jeffrey J. Wu, Nora H. Ruel, Sharon P. Wilczynski, Hanlin Gao, Maricela Covarrubias, Robert A. Figlin, Lawrence M. Weiss, Huiqing Wu

**Affiliations:** 1 Department of Molecular Medicine, City of Hope National Medical Center and Beckman Research Institute, Duarte, California, United States of America; 2 Department of Pathology, City of Hope National Medical Center and Beckman Research Institute, Duarte, California, United States of America; 3 Department of Information Sciences, City of Hope National Medical Center and Beckman Research Institute, Duarte, California, United States of America; 4 Department of Medical Oncology and Experimental Therapeutics, City of Hope National Medical Center and Beckman Research Institute, Duarte, California, United States of America; 5 Department of Cancer Biology, City of Hope National Medical Center and Beckman Research Institute, Duarte, California, United States of America; National Cancer Institute, National Institutes of Health, United States of America

## Abstract

Renal cell carcinoma (RCC) metastasis portends a poor prognosis and cannot be reliably predicted. Early determination of the metastatic potential of RCC may help guide proper treatment. We analyzed microRNA (miRNA) expression in clear cell RCC (ccRCC) for the purpose of developing a miRNA expression signature to determine the risk of metastasis and prognosis. We used the microarray technology to profile miRNA expression of 78 benign kidney and ccRCC samples. Using 28 localized and metastatic ccRCC specimens as the training cohort and the univariate logistic regression and risk score methods, we developed a miRNA signature model in which the expression levels of miR-10b, miR-139-5p, miR-130b and miR-199b-5p were used to determine the status of ccRCC metastasis. We validated the signature in an independent 40-sample testing cohort of different stages of primary ccRCCs using the microarray data. Within the testing cohort patients who had at least 5 years follow-up if no metastasis developed, the signature showed a high sensitivity and specificity. The risk status was proven to be associated with the cancer-specific survival. Using the most stably expressed miRNA among benign and tumorous kidney tissue as the internal reference for normalization, we successfully converted his signature to be a quantitative PCR (qPCR)-based assay, which showed the same high sensitivity and specificity. The 4-miRNA is associated with ccRCC metastasis and prognosis. The signature is ready for and will benefit from further large clinical cohort validation and has the potential for clinical application.

## Introduction

Renal cell carcinoma (RCC) accounts for about 3% of all malignant tumors in adults. Its worldwide incidence and mortality are approximately 209,000 and 102,000 per year respectively, including approximately 39,000 new cases and 13,000 deaths in the United States. [1] Clear cell RCC (ccRCC) represents the most common renal cancer histology, comprising 70–80% of all RCC cases. [2] About 30% of patients with newly diagnosed disease have evidence of metastases at presentation. [3] In the setting of metastasis, few patients achieve a durable remission with currently available therapies, with the response rate of about 15–25% and overall median survival of less than one year. [1] RCC metastasis cannot be reliably predicted based on patients’ clinical manifestations, pathologic findings or other currently available laboratory tests. Although several algorithms have been used to predict clinical outcome for patients with metastatic RCC (mRCC) on the basis of clinical and pathologic features, these do not incorporate the more complex biological features of individual patients. [2], Recent studies have shown that the metastatic capability of cancer is conferred by genetic changes occurring relatively early in tumorigenesis and that metastatic dissemination may occur continually throughout the course of primary tumor development. [5]–[7] In light of this, it is scientifically and clinically relevant to identify the metastasis-specific molecular biomarkers at the time of nephrectomy to predict ccRCC metastasis. The early identification of ccRCC metastatic potential may be beneficial for a more precise prediction of clinical outcomes and may ultimately be used to identify subsets of patients that may benefit from specific targeted therapies. [1].

MicroRNA (miRNA) is a group of small non-coding RNAs that regulate gene expression during development and differentiation. [8] Alteration of miRNA expression has been shown in malignancies [9]–[11] and plays a critical role in tumorigenesis and cancer progression [12]–[13]. Studies have specifically shown that certain miRNAs play important roles in various steps of the metastatic cascade, such as the epithelial-mesenchymal transition (EMT), adhesion, migration, invasion, apoptosis and angiogenesis. [14]–[15] Since one miRNA could regulate the expression of multiple genes, miRNA expression profiles can be more accurate in cancer subtyping than RNA profiles of protein-coding genes. [16]–[17] Molecular signatures based on miRNA expression have been shown to aid in diagnosis and prognostication of cancer. [18]–[19].

In this study, we used microarray technology to profile miRNA expression in benign kidney and ccRCC specimens. We analyzed the miRNA expression associated with metastasis in a training cohort to develop a 4-miRNA expression signature model that can determine the metastatic status and predict cancer-specific survival of ccRCC patients. More importantly, this molecular signature has been validated in an independent testing cohort and has also been converted to a quantitative PCR (q-PCR)-based assay. This study is ready for and will benefit from further large clinical cohort validation and has the potential to be applied in a routine clinical setting.

## Results

### Clinical Characterization of Patients’ Specimens in the Training and Testing Cohorts

A set of benign kidney specimens (n = 10) and a 28-sample ccRCC training cohort including localized (pT1, n = 13) and metastatic (M1, n = 15) tumor samples were used to profile miRNA expression in ccRCC and to develop a signature associated with metastasis. In addition, an independent testing cohort of primary tumors from 40 ccRCC patients was used to validate the signature. At the time of nephrectomy, these patients had stage I (pT1, n = 6), II (pT2, n = 5), III (pT3, n = 13) and IV (N2 or M1, n = 16) diseases**.** In the testing cohort patient group, 35 (35/40) patients had been followed for at least 5 years if no metastasis developed. At presentation, 16 (16/35) had concurrent metastasis and 13 (13/35) developed metastasis in the follow-up period, while 6 (6/35) did not have metastatic disease during the follow-up period. The clinical characteristics of the specimens are summarized in Table 1 and Table S1.

**Table 1:** Clinical characteristics of patients and tumor specimens (n = 68) in the training and testing cohorts.

		Training cohort numbers (%)	Testing cohort numbers (%)
**Patients/specimens**		28	40
**Age**	(mean±SD)	62.4±13.7	57.4±12.0
**Sex**	Male	15 (53.6)	24 (60.0)
	Female	13 (46.4)	16 (40.0)
**Grade** *	I	1 (7.7)	0 (0.0)
	II	8 (61.5)	12 (30.0)
	III	3 (23.1)	15 (37.5)
	IV	1 (7.7)	13 (32.5)
**Stage**	I	13 (46.4)	6 (15.0)
	II	0 (0.0)	5 (12.5)
	III	0 (0.0)	13 (32.5)
	IV	15 (53.6)	16 (40.0)
**Size** *	(mean±SD)	3.4±1.1	9.1±4.0

* The tumor grade and size are only applied to the primary tumors (n = 53).

### Profiling of miRNA Expression in ccRCCs

**Figure 1:**
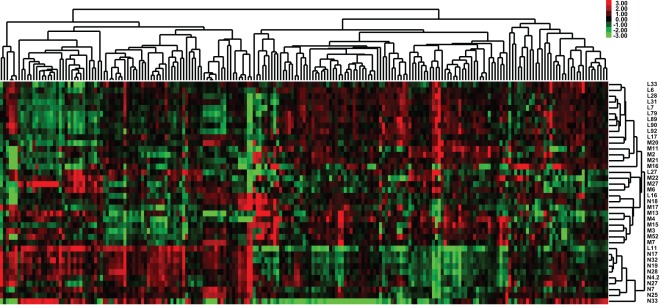
Unsupervised hierarchical clustering of miRNA expression levels using benign kidney and clear cell renal cell carcinoma (ccRCC) specimens. The miRNA expression levels were measured using the Agilent microarray technology with Quantile normalization and then filtered as described (see Material and methods). The transformed log2 intensities were mean centered across samples and a hierarchical clustering with average linkage was conducted with Cluster v3.0 and visualized with Java Treeview v1.1.3. (N-: benign kidney tissue; L-: T1 ccRCC specimen; M-: metastatic ccRCC specimen.).

Using the Agilent microarray technology, the miRNA expression of all of the benign kidney samples (n = 10) and the training cohort specimens (n = 28) was profiled. An unsupervised hierarchical clustering using these miRNA expression data could separate the benign and tumor samples (Figure 1). With a cut-off of a 2-fold change and FDR ≤0.05, 56 miRNAs were found to be aberrantly expressed in ccRCCs; 29 were up-regulated and 27 were down-regulated (Table 2). Within the tumor group, 21 miRNAs were found to be differentially expressed between localized and metastatic specimens; 7 were upregulated and 14 were down-regulated in the metastatic tumors (Table 3).

**Table 2:** Differentially expressed miRNAs in clear cell renal cell carcinoma compared to benign kidney tissue (n = 38).

miRNA ID	Benign	Tumor	Log2ratio	P value	FDR
hsa-miR-200c	11.03	6.91	−4.12	2.00E−05	3.76E−04
hsa-miR-141	12.90	8.81	−4.10	9.00E−05	8.87E−04
hsa-miR-122	3.03	6.90	3.87	9.00E−05	8.87E−04
hsa-miR-210	9.11	12.62	3.51	0.00E+00	0.00E+00
hsa-miR-514	6.71	3.72	−2.99	1.00E−03	5.05E−03
hsa-miR-224	5.02	7.98	2.96	1.00E−05	2.59E−04
hsa-miR-204	13.15	10.39	−2.77	7.64E−03	2.77E−02
hsa-miR-138	6.41	3.92	−2.49	7.80E−04	4.04E−03
hsa-miR-885-5p	3.87	6.22	2.35	1.34E−03	6.30E−03
hsa-miR-34b*	7.40	9.58	2.18	2.00E−05	3.76E−04
hsa-miR-30a*	12.89	10.73	−2.17	2.00E−05	3.76E−04
hsa-miR-7	5.07	7.17	2.11	5.00E−05	6.47E−04
hsa-miR-429	11.60	9.54	−2.06	2.60E−04	1.93E−03
hsa-miR-155	7.82	9.88	2.06	1.03E−03	5.08E−03
hsa-miR-144*	4.48	6.45	1.98	1.10E−02	3.55E−02
hsa-miR-142-5p	8.97	10.83	1.87	1.39E−02	4.20E−02
hsa-miR-30a	16.05	14.21	−1.85	4.00E-05	5.91E−04
hsa-miR-124	6.37	4.53	−1.84	1.17E−02	3.71E−02
hsa-miR-200b	13.19	11.36	−1.83	3.20E−04	2.07E−03
hsa-miR-454	5.91	7.72	1.81	2.80E−04	1.93E−03
hsa-miR-142-3p	11.64	13.43	1.80	7.61E−03	2.77E−02
hsa-miR-200a	12.37	10.67	−1.71	7.10E−04	3.93E−03
hsa-miR-939	10.18	8.50	−1.68	3.10E−04	2.07E−03
hsa-miR-886-3p	10.28	11.90	1.62	2.86E−03	1.21E−02
hsa-miR-130b	7.62	9.22	1.60	3.00E−05	5.18E−04
hsa-miR-532-3p	9.09	7.51	−1.58	1.20E−04	1.08E−03
hsa-miR-18a	6.10	7.69	1.58	7.40E−04	3.93E−03
hsa-miR-34a	11.76	13.3	1.54	0.00E+00	0.00E+00
hsa-miR-590-5p	7.57	9.11	1.54	3.60E−04	2.26E−03
hsa-miR-30c-2*	8.85	7.33	−1.52	7.40E−04	3.93E−03
hsa-miR-532-5p	10.40	8.91	−1.49	0.00E+00	0.00E+00
hsa-miR-340	7.24	8.72	1.49	2.60E−04	1.93E−03
hsa-miR-30c	13.59	12.12	−1.47	1.00E−05	2.59E−04
hsa-miR-30e*	10.83	9.43	−1.41	0.00E+00	0.00E+00
hsa-miR-139-5p	8.09	6.69	−1.40	9.06E−03	3.07E−02
hsa-miR-125a-3p	9.13	7.74	−1.39	1.19E−03	5.73E−03
kshv-miR-K12-3	10.17	8.79	−1.38	7.58E−03	2.77E−02
hsa-miR-30d	12.90	11.54	−1.36	5.40E−04	3.19E−03
hsa-miR-363	9.84	8.49	−1.35	6.00E−04	3.45E−03
hsa-miR-214	10.26	9.00	−1.26	1.27E−02	3.97E−02
hsa-miR-16	13.19	14.41	1.22	9.00E−05	8.87E−04
hsa-miR-10b*	7.85	6.63	−1.22	7.08E−03	2.77E−02
hsa-miR-362-5p	9.52	8.30	−1.21	1.60E−04	1.38E−03
hsa-miR-374a	9.65	10.86	1.21	8.28E−03	2.91E−02
hsa-miR-301a	8.63	9.80	1.17	1.60E−02	4.65E−02
hsa-miR-106b	11.72	12.86	1.15	1.00E−05	2.59E−04
hsa-miR-15a	12.38	13.53	1.15	4.00E−05	5.91E−04
hsa-miR-128	7.68	8.83	1.15	3.45E−03	1.40E−02
hsa-miR-93	9.72	10.86	1.14	8.00E−05	8.87E−04
hsa-miR-148a	11.14	12.27	1.13	2.59E−03	1.14E−02
hsa-miR-452	6.40	7.52	1.12	7.61E−03	2.77E−02
hsa-miR-425	8.22	9.32	1.10	8.00E−05	8.87E−04
hsa-miR-21	15.87	16.98	1.10	1.98E−03	9.11E−03
hsa-miR-663	7.37	6.27	−1.09	1.63E−02	4.69E−02
hsa-miR-15b	11.68	12.72	1.04	2.80E−04	1.93E−03
hsa-miR-23b	14.44	13.40	−1.03	1.80E−04	1.49E−03

Tumor: clear cell renal cell carcinoma.

**Table 3:** Differentially expressed miRNAs in metastatic clear cell renal cell carcinoma compared to localized tumor (n = 28).

miRNA ID	Localized	Metastatic	Log2 ratio	P value	FDR
hsa-miR-199b-5p	5.92	9.85	3.92	0.00E+00	0.00E+00
hsa-miR-204	12.04	8.95	−3.09	1.21E−03	1.57E−02
hsa-miR-489	8.25	5.84	−2.40	6.90E−04	1.02E−02
hsa-miR-139-5p	7.93	5.62	−2.30	0.00E+00	0.00E+00
hsa-miR-9*	4.17	6.26	2.09	6.69E−03	4.78E−02
hsa-miR-885-5p	7.31	5.27	−2.04	2.09E−03	1.98E−02
hsa-miR-10b*	7.53	5.85	−1.68	2.00E−05	1.38E−03
hsa-miR-10b	13.18	11.58	−1.60	3.20E−04	7.45E−03
hsa-miR-483-5p	6.61	8.14	1.54	1.75E−03	1.81E−02
hsa-miR-650	4.42	5.96	1.54	7.47E−03	4.87E−02
hsa-miR-575	8.25	9.77	1.52	2.10E−03	1.98E−02
hsa-miR-30c-2*	8.15	6.63	−1.51	1.00E−04	4.14E−03
hsa-miR-30a*	11.51	10.05	−1.46	5.50E−04	8.76E−03
hsa-miR-145	12.45	11.11	−1.34	3.87E−03	3.20E−02
hsa-miR-24-1*	7.82	6.49	−1.33	2.57E−03	2.31E−02
hsa-miR-200a	11.32	10.10	−1.23	7.76E−03	4.87E−02
hsa-miR-455-5p	9.04	7.84	−1.20	3.24E−03	2.79E−02
hsa-miR-130b	8.61	9.75	1.14	4.20E−04	7.76E−03
hsa-miR-145*	8.04	6.90	−1.14	5.96E−03	4.41E−02
hsa-miR-150*	5.85	6.96	1.11	4.55E−03	3.62E−02
hsa-miR-30a	14.77	13.72	−1.05	7.77E**-**03	4.87E−02

### Developing a 4-miRNA Signature Model for the Determination of the Status of ccRCC Metastasis

**Figure 2:**
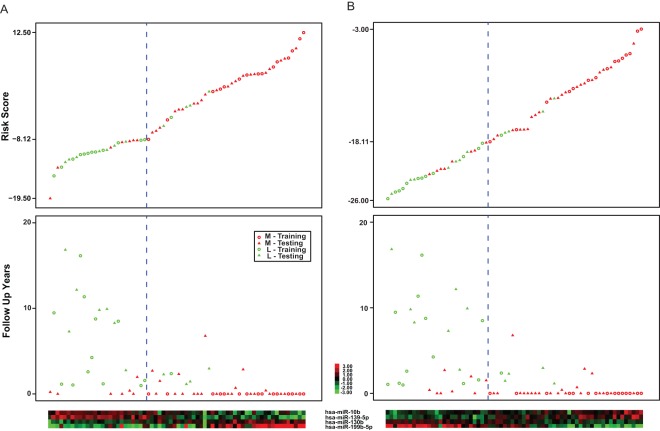
Clear cell renal cell carcinoma (ccRCC) metastasis-specific miRNA expression signature developed using microarray (A) and quantitative PCR (qPCR) (B). The risk scores for both training and testing cohort were calculated using the formula developed using microarray and quantitative PCR data of the training cohort. The risk score distribution (upper panel), survival status (middle panel) and expression profiles of the four miRNAs for all 68 ccRCC patients in the training and testing cohort are shown. The blue dotted line represents the signature cut-off to stratify patients into low and high risk groups. (M: metastatic patient; L: patient with no history of metastasis; Training: training cohort patient; Testing: testing cohort patient.).

Patients with stage I (T1) ccRCC usually have a favorable clinical outcome and their 5-year survival reaches 95% post nephrectomy. [20] In the study, T1 tumors were considered to be “good” tumors and were used to represent the control samples to compare with the metastatic ccRCCs. Using a univariate logistic regression test and Leave-One-Out cross validation (LOOCV) within the training set, the optimal p value cut-off to select the miRNAs associated with metastasis was determined. A range of p values were tested in this LOOCV test and the p value <0.01 was determined due to its best performance among all the p value cutoffs tested. Additionally, at least 2-fold change difference between the miRNA expression in metastatic and localized tumors was used to identify all the miRNAs that showed the largest difference between metastatic and local tumors. Four miRNAs, miR-10b, miR-139-5p, miR-130b and miR-199b-5p**,** satisfied the above criteria, and hence were selected to build a metastatic tumor signature. miR-199b-5p and miR-130b were over-expressed in metastatic tumors, while miR-10b and miR-139-5p were down-regulated (Figure 2A).

We used a risk score method to construct a signature model for ccRCC metastasis. [18] Specifically, the risk score formula is a linear combination of the expression levels of all the 4 miRNAs, weighted by the regression coefficients derived from the univariate logistic regression analysis, which is described as following: *Risk score = −1.275564×X_miR-10b_+2.106701×X_miR-130b_–2.278192×X_miR-139-5p_+1.101139×X_miR-199b-5p._*

**Figure 3:**
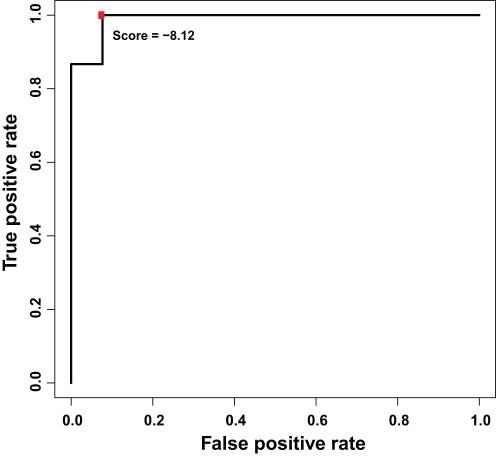
ROC curve of miRNA risk scores using microarray training data set. The red dot indicates the selected score cutoff of −8.12, which achieves the highest true positive rate (100%) and lowest false positive rate (8%).

The next step was to determine a cut-off point for a risk score to stratify patients into a group of high or low risk for metastasis. The risk score of each patient in the training set was calculated using the signature model developed, and the FPR and TPR within a range of cut-off scores were computed. The cut-off point of −8.12 was selected since it gave the best FPR and TPR (Figure 3). Therefore, a 4-miRNA signature model was developed to determine the risk status of tumor metastasis, in which a score ≥−8.12 indicates high risk.

### Validation of the 4-miRNA Signature in an Additional Independent Testing Cohort

**Figure 4:**
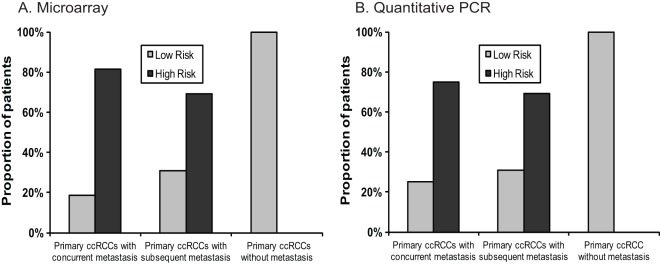
The 4-miRNA signature to predict high or low risk for metastasis using primary clear cell renal cell carcinoma (ccRCC) specimens in patients with concurrent (n = 16), subsequent (n = 13) and no (n = 6) metastasis. All the patients had been followed up for at least 5 years if no metastatic diseases reported. The signature was developed based on microarray (A) or quantitative PCR (qPCR) (B) data set.

To validate the signature, we used the independent testing cohort of primary ccRCCs. Each specimen was predicted to be either high or low risk based on its calculated risk score using the signature. The predicted risk status for each patient was then compared to the clinical outcome. Of 35 (35/40) patients with at least 5-year follow-up if no metastasis developed, 22 of 29 (22/29) that had metastatic disease had high risk primary tumors while 6 of 6 (6/6) with no metastasis had low risk tumors predicted by the signature. This gave a sensitivity of 76% and a specificity of 100%. Specifically, 13 of 16 patients (81%) with concurrent metastasis were predicted to be of high risk; 9 of 13 (69%) with subsequent metastasis, including 2 of 5 (2/5, 40%) with T1/2 tumors and 8 of 9 (8/9, 89%) with T3 tumors, were predicted to be of high risk; and 6 of 6 (100%) without metastasis were predicted to be of low risk (Figure 4A). For all 40 patients with or without 5-year follow-up, the signature showed a sensitivity of 76% (22/29) and a specificity of 64% (7/11). If patients with concurrent metastasis (stage IV) were not included, the sensitivity was 69% (9/13) and the specificity remained the same (7/11, 64%). In the additional 5 primary ccRCC specimens, 1 (1/5) was predicted to be of low risk and 4 (4/5) was predicted to be of high risk. However, these specimens were collected within the last two years, and whether these patients will develop metastasis is not known. Interestingly, all 4 patients predicted to have high risk had stage III diseases and the 1 predicted to have low risk had stage I disease.

The risk score of each ccRCC specimen determined by the 4-miRNA signature model is associated with the status of metastasis (OR = 5.50, 95% CI = 1.23–24.51, p<0.05). Other varieties, such as a patient’s sex, age, tumor grade and stage, did not reliably predict metastasis (Table 4).

**Table 4:** Relative odds for patients with metastasis associated with the risk core, patient’s age and sex, tumor grade and size, and clinical stage in the testing cohort (n = 40).

	Met*	Non-met*	OR	95% CI	P value
**Risk Score**					
Microarray					
Score ≤−8.12	7	7	–	–	–
Otherwise	22	4	5.50	1.23–24.51	0.03
Quantitative PCR					
Score ≤−18.11	8	7	–	–	–
Otherwise	21	4	4.59	1.05–20.06	0.04
**Age**					
≤50	8	2	–	–	–
51–60	7	5	0.35	0.05–2.41	0.29
61–70	9	3	0.75	0.10–5.69	0.78
>70	4	2	0.50	0.05–4.98	0.55
**Sex**					
Female	10	6	–	–	–
Male	18	6	1.80	0.46–7.09	0.40
**Grade**					
II	6	6	–	–	–
III	11	4	2.75	0.55–13.75	0.22
IV	11	2	5.50	0.84–36.20	0.08
**Size**					
≤4	2	1	–	–	–
>4–≤7	6	7	0.43	0.03–5.99	0.53
>7	20	4	2.50	0.18–34.67	0.50
**Stage** **					
I&II	5	6	–	–	–
III	8	5	1.92	0.38–9.80	0.43
IV	16	0	20.65	2.52–∞	<0.01

* Met: patient with concurrent and subsequent metastasis; Non-met: patient without metastasis.

** Exact logistic regression is used due to o count in stage IV nom-met patients.

### The 4-miRNA Signature Correlates with Overall Cancer-specific Survivals

**Figure 5:**
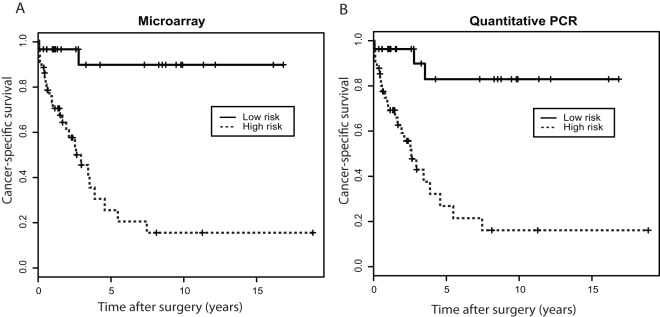
Kaplan-Meier analysis of cancer-specific survival in clear cell renal cell carcinoma (ccRCC) patients (all the training and testing cohort patients, n = 68) stratified by the 4-miRNA signature using microarray (A) and quantitative PCR (qPCR) data.

We were also interested in examining whether this signature model could be independently associated with the cancer-specific survival. With patients in the combined training and testing cohorts (n = 68), a univariate Cox regression analysis showed that the predicted risk status was a significant prognostic factor for the patient’s cancer-specific survival (Table 5). The relative risk for patients predicted to be of high risk was 12.68 compared to patients of low risk (HR = 12.68, 95% CI = 2.97−54.13, p<0.0001). The stage of disease was the only other significant prognostic factor, while age, sex, tumor grade and size were not correlated with survival. Patients predicted to be of high risk had a 5-year survival rate of only 32%, whereas those of low risk had a 5-year survival rate of 84% (Figure 5A).

**Table 5:** Univariate Cox regression analysis of all patients (n = 68).

	HR	95% CI	P value (Wald test)
**Risk status** **(high vs low)**			
microarray	12.68	2.97–54.13	<0.0001
quantitative PCR	8.80	2.62–29.58	<0.0001
**Age**			
51–60 vs ≤50	1.35	0.50–3.66	0.56
61–70 vs ≤50	0.89	0.31–2.56	0.84
>70 vs ≤50	0.54	0.11–2.60	0.44
Age (continuous)	0.98	0.95–1.01	0.17
Sex (male vs female)	1.70	0.74–3.91	0.21
**Grade** *			
III vs I&II	2.67	0.80–8.89	0.11
IV vs I&II	2.52	0.67–9.42	0.17
**Stage**			
III vs I&II	4.39	0.72–26.53	0.11
IV vs I & II	20.23	4.66–87.79	<0.0001
**Size** *			
4–7 vs ≤4	4.36	0.51–37.31	0.18
>7 vs ≤4	6.93	0.89–53.84	0.06

* The tumor grade and size are only applied to the primary tumors.

### Converting the Microarray-based Signature to a RT-PCR Based Assay

The greatest challenge for performing RT-PCR based tissue miRNA expression analysis is to find a reliable reference miRNA or small RNA for the test normalization. To further develop a 4-miRNA signature assay using a RT-PCR platform, the microarray database of miRNA expression in all of the benign and tumor kidney samples (n = 78) was analyzed. miR-24 was found to be the most stably expressed in all of the samples (Table 6). Therefore, miR-24 was selected as a reference miRNA for normalization.

**Table 6:** Top 10 miRNAs with the least CV in expression of tumor and benign kidney tissue (n = 78).

miRNA ID	SD	Mean	CV
hsa-miR-24	0.49	14.29	3.43%
hsa-miR-27a	0.54	14.32	3.77%
hsa-miR-26a	0.53	13.93	3.80%
hsa-miR-21	0.68	16.93	4.02%
hsa-miR-23a	0.62	14.61	4.24%
hsa-miR-30b	0.59	13.15	4.49%
hsa-miR-103	0.60	12.86	4.67%
hsa-miR-331-3p	0.53	11.13	4.76%
hsa-miR-29a	0.72	14.75	4.88%
hsa-miR-23b	0.71	13.35	5.32%

SD: standard deviation; CV: coefficient of variation.

Each of the 4 miRNAs selected for the signature in each specimen in the training and testing cohorts were used, and their expression, normalized by that of miR-24, was analyzed using ABI TaqMan MicroRNA Assay. Similar to the microarray study described, using the training cohort, a PCR-based risk score formula model (*Risk score* = *1.431559*×*X_miR-10b_*–*1.530509×X_miR-130b_*+*1.888144*×*X_miR-139-5p_–2.569280*×*X_miR-199b-5p_*) was constructed and the corresponding high risk score cut-off (−18.11) was determined (Figure 2B). The signature was then validated using the testing cohort. For the 35 primary tumor cases with follow-up, the sensitivity and specificity of the qPCR-based signature were 72% (21/29) and 100% (6/6). Among these cases, 12 of 16 (75%) with concurrent metastasis were predicted to be of high risk; 9 of 13 (69%) with subsequent metastasis, including 2/5 (40%) T1/2 and 8/9 (89%) tumors, were predicted to be of high risk; and 6 of 6 (100%) without metastasis were predicted to be of low risk (Figure 4B). For all 40 cases with or without follow-up, the overall sensitivity and specificity were 72% (21/29) and 64% (7/11). The signature was also found to be significantly associated with cancer-specific survival (HR = 8.8, 95% CI = 2.62−29.58, p<0.0001) (Figure 5B, Table 5).

## Discussion

Generally, mRCC has an extremely poor prognosis. [1] Early identification of patients with high risk for cancer metastasis can enhance disease outcome prediction, stratify patients for suitable treatment and potential clinical trials and, ultimately, decrease cancer-specific mortality.

miRNA plays important roles in tumorigenesis and progression. Many miRNAs reported to be dysregulated in RCC were also seen in our current study. [21]– Studies of cancer metastasis have shown that certain miRNAs, termed “metastamir”, were specifically involved in the critical steps of the metastatic cascade and appeared to be either pro-metastatic or anti-metastatic by regulating their target genes. [15] In the current study, we identified and used the altered expression of miR-10b, miR-130b, miR-139-5p and miR-199b-5p to generate a metastasis-specific signature. miR-139-5p is down-regulated in endometrial serous and gastric adenocarcinoma. [19], [25] Overexpression of miR-130b is involved in the growth control of breast epithelial cells via the modulation of the cell cycle inhibitor p21^Waf1/Cip1^. [26] Altered expression of miR-199b-5p is associated with *HES-1* gene regulation and metastatic spread of medulloblastoma. [27] Dysregulation of miR-10b has been observed in malignant glial tumors, esophageal cancer cell lines and primary breast cancer, though whether it is involved in breast metastasis was in debate. [28]–[32] In our study, miR-10b has been found to be down-regulated in ccRCCs. The expression appears to be even lower in metastatic ccRCCs than that in localized non-metastatic tumors. Our preliminary data revealed that the overexpression and knockdown of miR-10b in a cell line derived from a metastatic ccRCC caused decrease and increase in proliferation and invasion of tumor cells, respectively (data not shown), which might be involved in regulating CDK6 and other target genes (www.miRBase.com and www.oncomine.org).

**Figure 6:**
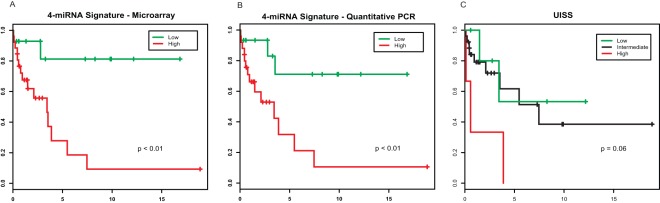
Kaplan-Meier analysis of cancer-specific survival in clear cell renal cell carcinoma (ccRCC) patients. They are all the testing cohort patients who are stratified by the 4-miRNA signature using microarray (n = 40) (A) and quantitative PCR (qPCR) (n = 40) (B), and by the UCLA Integrated Staging System (UISS) (n = 35) (C). (low: low risk group; high: high risk group; intermediate: intermediate risk group.).

**Table 7:** Univariate Cox regression analysis of patients in the testing cohort (n = 40).

	HR	95% CI	P value (Wald test)
**Risk status (high vs low)**			
microarray	6.81	1.52–30.52	0.01
quantitative PCR	4.88	1.37–17.38	0.01
**UISS risk status** *			
high vs low	5.60	0.90–34.88	0.07
intermediate vs low	1.45	0.31–6.77	0.63

* Five patients’ UISS risk scores were not available (n = 35).

The 4-miRNA signature is associated with ccRCC metastasis. Though the validation test has shown that the signature appeared to be more powerful in identifying concurrent metastases (81%), the signature is also significantly associated with subsequent/future metastasis of the primary tumors (69%), including patients with T3 (89%) and T1/2 (40%) diseases. Patients with stage I or II ccRCCs usually have 5-year survival of 95% and 88% and are often less likely to develop metastasis compared to those with late stage diseases. [10] Clinically, it is extremely helpful if the metastatic potential of T3 tumors can be predicted early, ideally at the time of nephrectomy. Due to the limitation of sample size, the signature model warrants and will benefit from further large cohort validations.

Currently, there is no clinically available molecular assay to predict ccRCC metastasis. A retrospective study reported that *IMP3* expression analysis by immunohistochemistry could predict RCC metastasis and prognosis. [33] The study identified IMP3-positive tumors in 59/95 metastatic RCCs, 60/119 primary RCCs with metastasis and 11/287 primary RCCs with no metastasis, rendering an overall sensitivity of 56%, specificity of 96% and a hazard ratio of 5.66. Our 4-miRNA signature achieves a higher sensitivity (76% in overall and 69% for predicting tumor with future metastasis), specificity (100%) and hazard ratio (12.68) as compared to the IMP3 study. However, our current study has fewer cases tested and is only limited to the clear cell type of RCCs. We are planning to evaluate our signature model using much larger cohorts and to test the effectiveness of the current model for other types of RCCs.

Our signature has also shown its association with disease prognosis. Currently, the UCLA Integrated Staging System (UISS) is a widely used prognostic tool for RCC patient’ outcome. It classifies cases into high, intermediate and low risk groups, based on tumor stage, histological grade and Eastern Cooperative Oncology Group (ECOG) performance status (PS). [34] As reported in international multi-center studies [34]–[35], the overall 5-year cancer-specific survival rates estimated by the UISS were 92–94%, 65–78% and 30–48% for the low, intermediate and high risk group patients, respectively. To compare the UISS with our signature, we assigned a UISS risk score to the 35 of 40 testing cohort cases with available information of ECOG performance status. The predicted 5-year cancer-specific survival rates were 0%, 63% and 52% for the high, intermediate and low risk group patients, respectively, by UISS, compared to 32% and 84% for the high and low risk patients, respectively, by the 4-miRNA signature (Figure 6). The UISS score seems not to be a significant prognostic factor for the cases tested in our testing cohort. However, there are only 35 cases tested, this finding might not be representative. Our risk scores based on both microarray and RT-PCR are statistically significant (Table 7). The hazard ratio of our high versus low risk status is 6.81 (95% CI = 1.52−30.53, p value <0.01) and 4.88 (95% CI = 1.37−17.38, p value <0.01), by microarray and qPCR, respectively.

In the study, we have found that the clinical stage was the only other significant prognostic factor. Patients’ age, sex, tumor grade and size have not been found to be significantly correlated with survival using our data. As mentioned previously, the sample size of our study is relatively small and further large cohort validation is definitively needed for more accurate analysis.

The miRNA signature developed from the current study has the potential to be applied in a routine clinical setting. Certainly, converting a microarray-derived signature to a PCR-based test will make the signature assay more practical for a clinical laboratory usage. It is always very challenging to perform qPCR-based miRNA expression studies in clinical tissues, mainly because there have been no reliable conventionally known or commercially available reference miRNAs or other small RNAs to serve as house-keeping genes in mRNA expression studies. This probably explains why many published PCR-based clinical tissue studies of miRNA expression are not reproducible. It has been suggested to use miR-191 and miR-103 for tissue miRNA normalization. [36] However, miRNA expression is very tissue-specific. [8] Some miRNAs stably expressed in certain tissue types might be expressed differently in other tissue types. Having carefully analyzed our own microarray data, we found that miR-24 is most constantly and stably expressed among all the benign and malignant kidney specimens. The 4-miRNA signature based on qPCR data also showed a high sensitivity (72%) and specificity (100%), as well as a similar association with cancer-specific death, which further validated our microarray results and provided the technologic basis for a possible larger scale qPCR-based validation. Our recent study has shown that formalin-fixed paraffin-embedded (FFPE) samples can be reliably used for miRNA expression profiling studies. [24] We are planning to validate the signature developed in the current study using a larger FFPE tissue cohort and to evaluate it in the context of specific therapies.

## Materials and Methods

### Tissue Sample Preparation and Total RNA Extraction

A total of 78 frozen benign kidney and ccRCC specimens were used for the study. All the samples were selected from the frozen tissue specimens stored at the City of Hope National Medical Center (COH) Tumor Bank. All the available frozen specimens collected from ccRCC patients at COH between 1986 and 2008 which were included for the study were first tested using the 2100 Bioanalyzer (Agilent Technologies, Inc., Santa Clara CA) as quality control (total RNAs with the quality index >5.0 for each specimen).

Specifically, the benign kidney tissue and primary tumors were sampled from the nephrectomy specimens. All the qualified benign samples (n = 10) were randomly selected from the available specimen collection. All the localized ccRCC samples (n = 13) for the training cohort were pT1 (stage I) primary tumors with no reported subsequent metastasis were randomly selected from the same available collection. The metastatic ccRCC samples (n = 15) used for the training cohort included all the available frozen specimens taken from the resection/biopsy specimens of distant metastases or lymph nodes during the time period. All the remaining available primary ccRCC specimens were used for the testing cohort (n = 40). The information of the patients’ age, sex, race and clinical tumor stage is listed in a table (see Table S1).

The samples were snap-frozen shortly after operation and had been stored at –80°C at the COH Tumor Bank. The protocol for using these samples was approved by the COH Cancer Protocol Review and Monitoring Committee (CPRMC) and Institutional Review Board (IRB). A waiver of informed consent and HIPAA authorization has also been approved by the COH IRB. Total RNA was extracted from up to 10 sections (10 µm in thickness) of each sample as described previously. [24].

### Microarray Analysis for miRNA

Microarray testing of miRNA expression was performed at the COH Microarray Core using the Agilent human miRNA microarray V2 (Agilent Technologies, Inc., Santa Clara CA), which contains probes for 723 human miRNAs from Sanger miRBase 10.1, as described previously. [24] Briefly, 1 µg total RNA was labeled with Cy3 with T4 RNA ligase and hybridized to the array for 20 hours at 55°C. The arrays were then washed and scanned using an Agilent scanner with default settings. Scanned images were subject to Agilent Feature Extraction Software v. 10.5 for raw data processing.

### Statistical Analysis and the Method of miRNA Signature Construction

The analysis was performed using R statistical language. Raw data from Agilent miRNA array was processed by Quantile normalization, followed by log2 transformation with an offset of 1. [37] Differentially expressed miRNAs between tumor (training cohort) and benign samples were selected using t-test with a FDR ≤0.05 and fold change of 2.

To develop the miRNA signature, univariate logistic regression analysis was used to identify miRNAs that were associated with metastasis. Specifically, the miRNA signature development consists of the following steps: 1) Univariate logistic regression analysis was used to identify miRNAs that were associated with metastasis; 2) A mathematical formula based on the expression levels of the identified miRNAs was developed to assign a risk score for each patient; 3) A risk score cut-off was determined to classify each patient into a high or low risk group.

Step 1 is a feature selection step, and step 2 and 3 are model building steps. In step 1, a range of p values (0.05, 0.02, 0.01, 0.005, 0.002 and 0.001) were tested with LOOCV and found the best p value cutoff of 0.01. Specifically, at each iteration step of the cross validation, one sample was tested (the test sample) while the others remained in the training group (n = 28–1). During the process, the feature selection and formula development were repeated within each iteration step and the signature model was used to predict the status of the test sample. Following Simon et al’s suggestion [38], the feature selection and signature model building steps were entirely independent of the test sample. This is critical to ensure that the performance of the signature model formula developed can be estimated without any bias. Using LOOCV, we could achieve a minimal error rate with a p value <0.01. We also arbitrarily required a fold-change between metastatic and localized specimens of ≥2, which could help to develop a PCR-based assay for the signature. These criteria resulted in 4 miRNAs that were significantly associated with metastasis.

To investigate the effectiveness of these four miRNAs as a signature to determine the status of metastasis, a mathematical formula constructed, taking into account both the strength and the positive or negative association of each miRNA with metastasis. More specifically, a risk score was calculated for each patient in the training cohort group using the formula, which was a linear combination of the expression levels of the miRNAs, weighted by the regression coefficients derived from the aforementioned univariate logistic regression analysis. To choose the optimal risk score cutoff, a range of scores were tested to stratify these patients into high and low risk groups. The false positive rate (FPR) and true positive rate (TPR) of these cutoffs were calculated and a risk score cutoff point was selected based on the lowest FPR and highest TPR (FPR = 8%, TPR = 100%) (Figure 3). Therefore, a miRNA signature model, which consists of a risk score formula and a high risk score cutoff, was developed to classify patients into high and low risk groups for developing metastasis.

The performance of the signature was further validated using the additional independent testing cohort data set (n = 40), in which each patient’s risk for developing metastasis was determined based on the calculated risk score and then compared to the clinical follow-up information.

To investigate whether the 4-miRNA signature was also an independent prognostic factor for cancer specific survival, univariate Cox regression analysis was used to examine the patients’ risk status based on the signature, patient age and gender, tumor histologic grade and size, clinical stage and available UISS score (see discussion). A p value <0.05 was used to determine significance.

### RT-PCR Testing

In each sample, the expression of hsa-miR-10b, 130b, 139–5p and 199b-5p was analyzed using RT-PCR TaqMan MicroRNA Assays and 7900 HT Fast Real-time PCR System (Applied Biosystems, Carlsbad, CA). Briefly, 10 ng of total RNA from each sample was subjected to reverse-transcription forming 1st strand cDNA with mature miRNAs specific primers containing stem loop, followed by real-time PCR with TaqMan probes. PCR reactions for each sample were carried out in triplicate. Each miRNA expression, normalized by hsa-miR-24, was quantified using the formula *X = 2^–ΔCT^*, where *ΔCT = CT_(miR-X)_–CT_(miR-24)_*.

## Supporting Information

Table S1 **Patients’ Information.**
(DOC)
