# Supplementary material for: Identification of a 4-microRNA Signature for Clear Cell Renal Cell Carcinoma Metastasis and Prognosis
Source: PLoS One. 2012 May 18;7(5):e35661. doi: 10.1371/journal.pone.0035661 (PMC3356334; doi:10.1371/journal.pone.0035661)
Supplement: Supplementary file 1 [file pone.0035661.s001.doc]

## Sheet: Sheet1

Table S1. Patients' Information
Training cohort
ID	Age	Sex	Race	Tumor grade	Tumor stage	Tumor status
L6	45	M	white	2	I	Localized tumor
L7	56	F	white	2	I	Localized tumor
L11	60	M	white	2	I	Localized tumor
L16	69	F	white	2	I	Localized tumor
L17	66	F	white	2	I	Localized tumor
L27	80	F	white	2	I	Localized tumor
L28	84	F	white	2	I	Localized tumor
L31	66	F	white	2	I	Localized tumor
L33a	55	F	white	1	I	Localized tumor
L79	64	F	white	3	I	Localized tumor
L89	76	M	white	3	I	Localized tumor
L90	83	M	white	4	I	Localized tumor
L92	75	M	white	3	I	Localized tumor
M2	58	M	white		IV	Metastatic tumor
M3	69	M	white		IV	Metastatic tumor
M4	83	M	white		IV	Metastatic tumor
M6	63	F	white		IV	Metastatic tumor
M7	76	M	white		IV	Metastatic tumor
M11	54	M	white		IV	Metastatic tumor
M13	56	M	white		IV	Metastatic tumor
M15	53	M	Asian		IV	Metastatic tumor
M16	45	F	white		IV	Metastatic tumor
M17	43	M	white		IV	Metastatic tumor
M20	62	F	white		IV	Metastatic tumor
M21	54	M	white		IV	Metastatic tumor
M22	31	F	Hispanic		IV	Metastatic tumor
M27	75	M	white		IV	Metastatic tumor
M52	45	F	white		IV	Metastatic tumor
Testing cohort
ID	Age	Sex	Race	Tumor grade	Tumor stage	Metastatic status
P1	65	F	Asian	2	I	No metastasis
P2	75	M	white	2	I	No metastasis*
P3	48	M	white	2	I	Subsequent metastasis
P4	61	M	white	2	I	Subsequent metastasis
P5	60	M	white	2	II	Subsequent metastasis
P6	36	M	white	4	II	Subsequent metastasis
P7	49	M	Hispanic	4	II	No metastasis
P8	37	F	white	2	I	No metastasis
P9	74	F	white	2	II	No metastasis
P10	53	M	white	3	IV	Concurrent metastasis
P11	47	F	white	2	II	Subsequent metastasis
P12	58	M	white	3	I	No metastasis
P13	67	F	white	4	III	Subsequent metastasis
P14	51	M	white	3	III	No metastasis*
P15	62	F	white	2	III	No metastasis*
P16	69	M	white	3	III	Subsequent metastasis
P17	67	M	white	2	III	Subsequent metastasis
P18	59	F	white	3	III	No metastasis
P19	69	F	white	3	III	Subsequent metastasis
P20	53	M	white	4	III	No metastasis*
P21	52	M	white	2	III	No metastasis*
P22	61	M	white	2	III	Subsequent metastasis
P23	62	F	Hispanic	4	III	Subsequent metastasis
P24	52	F	white	3	IV	Concurrent metastasis
P25	51	F	white	4	IV	Concurrent metastasis
P26	63	M	white	3	III	Subsequent metastasis
P27	71	F	black	3	III	Subsequent metastasis
P28	22	M	white	4	IV	Concurrent metastasis
P29	60	M	white	3	IV	Concurrent metastasis
P30	72	M	Asian	3	IV	Concurrent metastasis
P31	60	M	white	4	IV	Concurrent metastasis
P32	36	F	white	4	IV	Concurrent metastasis
P33	72	M	Asian	4	IV	Concurrent metastasis
P34	57	F	white	3	IV	Concurrent metastasis
P35	50	M	white	4	IV	Concurrent metastasis
P36	45	F	white	3	IV	Concurrent metastasis
P37	75	M	white	4	IV	Concurrent metastasis
P38	64	F	white	3	IV	Concurrent metastasis
P39	47	M	other	3	IV	Concurrent metastasis
P40	64	M	white	4	IV	Concurrent metastasis
*Without 5 years of follow-up.
